# Monodisperse α-Fe_2_O_3_ Mesoporous Microspheres: One-Step NaCl-Assisted Microwave-Solvothermal Preparation, Size Control and Photocatalytic Property

**DOI:** 10.1007/s11671-010-9742-7

**Published:** 2010-08-18

**Authors:** Shao-Wen Cao, Ying-Jie Zhu

**Affiliations:** 1 State Key Laboratory of High Performance Ceramics and Superfine Microstructure, Shanghai Institute of Ceramics, Chinese Academy of Sciences, 200050, Shanghai, People's Republic of China

**Keywords:** α-Fe_2_O_3_, Microwave, Mesoporous, Microsphere, Photocatalysis

## Abstract

A simple one-step NaCl-assisted microwave-solvothermal method has been developed for the preparation of monodisperse α-Fe_2_O_3_ mesoporous microspheres. In this approach, Fe(NO_3_)_3_ · 9H_2_O is used as the iron source, and polyvinylpyrrolidone (PVP) acts as a surfactant in the presence of NaCl in mixed solvents of H_2_O and ethanol. Under the present experimental conditions, monodisperse α-Fe_2_O_3_ mesoporous microspheres can form via oriented attachment of α-Fe_2_O_3_ nanocrystals. One of the advantages of this method is that the size of α-Fe_2_O_3_ mesoporous microspheres can be adjusted in the range from ca. 170 to ca. 260 nm by changing the experimental parameters. High photocatalytic activities in the degradation of salicylic acid are observed for α-Fe_2_O_3_ mesoporous microspheres with different specific surface areas.

## Introduction

The fabrication of mesoporous materials of transition metal oxides has attracted more and more attention in recent years for their unique catalytic, electrochemical, magnetic and adsorptive properties [1-4]. Among them, α-Fe_2_O_3_ mesoporous materials are of particular interest, because α-Fe_2_O_3_ is widely used in catalysis [5], photoelectrodes [6], sensors [7], the anode material for Li-ion batteries [8] and so on. As an important n-type semiconductor, α-Fe_2_O_3_ is also used as a photocatalyst [9,10], especially in the degradation of salicylic acid [11-13]. Salicylic acid is a complexing agent that forms stable complexes with iron ions, and it is one of pollutants in waste effluent [14]. Mesoporous structures will benefit the photocatalytic activity of α-Fe_2_O_3_ due to the high specific surface area and the redox activity of the surfaces and nanopores.

Although the preparation of mesoporous silica, aluminosilicates, aluminophosphates and related materials is already well established [15-18], however, the synthesis of mesoporous materials of transition metal oxides is much more difficult and less reported [19,20]. Several mesoporous materials of transition metal oxides such as TiO_2_, ZrO_2_, Nb_2_O_5_, WO_3_ and MnO_x_[21-27] have been prepared owing to researchers' unremitting effort. α-Fe_2_O_3_ mesoporous structures were prepared using soft templating methods [1,28-31], as well as using mesoporous silica as hard template [19]. However, such methods suffer from some disadvantages. Soft templating methods usually lead to the formation of mesoporous α-Fe_2_O_3_ with amorphous walls, while the hard templating methods usually involve multistep processes and sometimes lead to the damage of pore structures during the removal of hard templates.

Monodisperse nanocrystals display novel properties thus to stimulate intensive researches on the synthesis of monodisperse nanocrystals for their fundamental and technological importance [32]. However, challenges still arise, how to combine the mesoporous structure with monodisperse microspheres, for the enhancement of the structural stability and photocatalytic property of α-Fe_2_O_3_. Herein, we report a simple one-step NaCl-assisted microwave-solvothermal method for the preparation of monodisperse α-Fe_2_O_3_ mesoporous microspheres. In the present approach, monodisperse α-Fe_2_O_3_ mesoporous microspheres can form via oriented attachment of α-Fe_2_O_3_ nanocrystals in the presence of NaCl. One of the advantages of this method is that the size of α-Fe_2_O_3_ mesoporous microspheres can be adjusted in the range from ca. 170 to ca. 260 nm by changing the experimental parameters. High photocatalytic activities in the degradation of salicylic acid are observed for typical samples of α-Fe_2_O_3_ mesoporous microspheres with different specific surface areas.

## Materials and Methods

### Preparation of Monodisperse α-Fe_2_O_3_ Mesoporous Microspheres

In a typical synthetic procedure, 0.404 g Fe(NO_3_)_3_ · 9H_2_O, 0.117 g NaCl and 0.111 g PVP (K-30) were dissolved in mixed solvents of 15 ml H_2_O and 15 ml ethanol under magnetic stirring. The resultant solution was loaded into a 60-ml Teflon autoclave, sealed, microwave-heated to 120°C and kept at this temperature for 30 min. The microwave oven used for sample preparation was microwave-solvothermal synthesis system (MDS-6, Sineo, Shanghai, China). After cooled to room temperature, the products were collected and washed by centrifugation–redispersion cycles with deionized water and alcohol three times, respectively. Please refer to Table 1 for the detailed preparation conditions for typical samples.

**Table 1:** Experimental parameters for the preparation of typical samples by the microwave-solvothermal method

Sample no.	Solution	Temperature (°C)	Time (min)	Size (nm)
1	0.404 g Fe(NO_3_)_3_ · 9H_2_O + 0.117 g NaCl + 0.111 g PVP + 15 ml H_2_O + 15 ml ethanol	120	30	ca. 170
2	0.404 g Fe(NO_3_)_3_ · 9H_2_O + 0.111 g PVP + 15 ml H_2_O + 15 ml ethanol	120	30	/
3	0.404 g Fe(NO_3_)_3_ · 9H_2_O + 0.117 g NaCl + 15 ml H_2_O + 15 ml ethanol	120	30	/
4	0.404 g Fe(NO_3_)_3_ · 9H_2_O + 0.117 g NaCl + 0.222 g PVP + 15 ml H_2_O + 15 ml ethanol	120	30	ca. 205
5	Same as sample 1	120	60	ca. 225
6	Same as sample 1	140	30	ca. 260

### Photocatalytic Activity Measurements

The photocatalytic reactor consisted of two parts: a 70-ml quartz tube and a high-pressure Hg lamp. The Hg lamp was positioned parallel to the quartz tube. In all experiments, the photocatalytic reaction temperature was kept at about 35°C. The reaction suspension was prepared by adding the sample (20 mg) into 50 ml of salicylic acid solution with a concentration of 20 mg l^-1^. The suspension was sonicated for 15 min and then stirred in the dark for 30 min to ensure an adsorption/desorption equilibrium prior to UV irradiation. The suspension was then irradiated using UV light under continuous stirring. Analytical samples were withdrawn from the reaction suspension after various reaction times and centrifuged at 10,000 rpm for 5 min to remove the particles for analysis.

### Characterization of Samples

The as-prepared samples were characterized using X-ray powder diffraction (XRD) (Rigaku D/max 2550V, Cu K*α* radiation, λ = 1.54178 Å), scanning electron microscopy (SEM) (JEOL JSM-6700F) and transmission electron microscopy (TEM) (JEOL JEM-2100F). The Brunauer–Emmett–Teller (BET) surface area and pore size distribution were measured with an accelerated surface area and porosimetry system (ASAP 2010, USA). The photocatalytic reactions were carried out under irradiation of a 300-W high-pressure Hg lamp (GGZ300, Shanghai Yaming Lighting) with a maximum emission at about 365 nm. The salicylic acid concentrations were analyzed using a UV–vis spectrophotometer (UV-2300, Techcomp) at a wavelength of 297 nm.

## Results and Discussion

The detailed preparation procedures for the samples are described in the experimental section, and the preparation conditions for typical samples are listed in Table 1.

**Figure 1:**
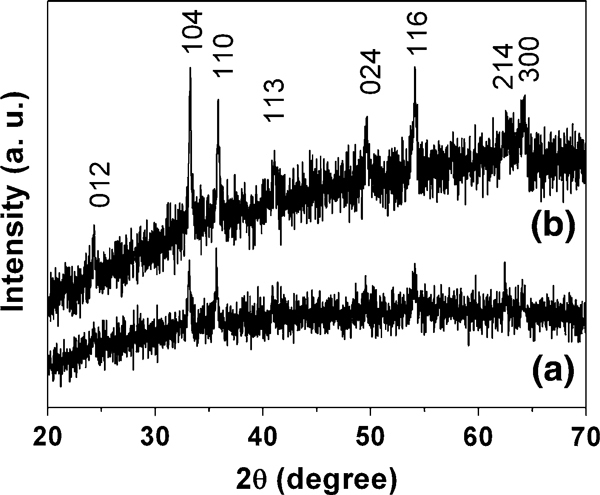
**XRD patterns: *a* sample 1; *b* sample 6**.

Figure 1a shows the XRD pattern of sample 1 prepared using 0.404 g Fe(NO_3_)_3_ · 9H_2_O, 0.117 g NaCl and 0.111 g PVP in mixed solvents of 15 ml H_2_O and 15 ml ethanol by microwave-solvothermal method at 120°C for 30 min. Based on the analysis of the XRD pattern in Figure 1a, the product is α-Fe_2_O_3_ with a hexagonal structure (JCPDS No. 80-2377).

**Figure 2:**
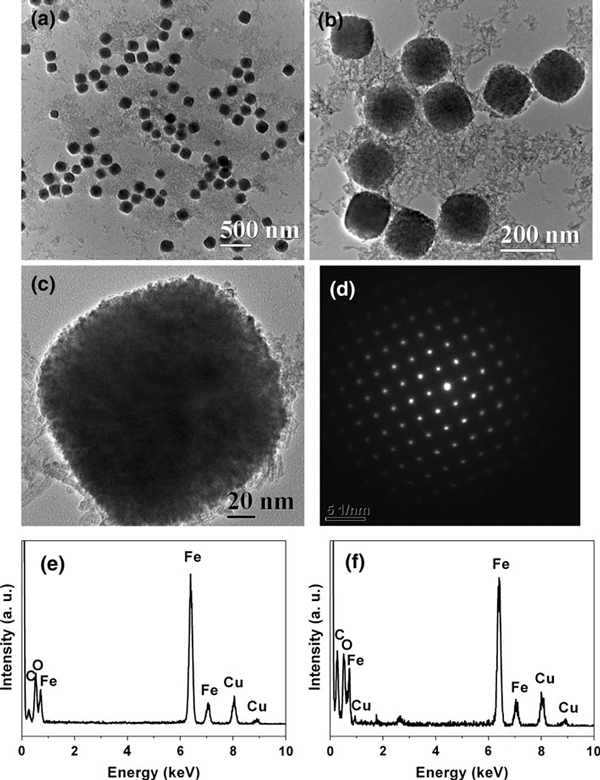
**Characterization of sample 1: a–c TEM micrographs; d the SAED pattern of a single microsphere; e EDS spectrum of the microspheres; f EDS spectrum of dispersed nanocrystals**.

TEM micrographs were recorded to investigate the morphology and structure of sample 1, as shown in Figure 2a–c. One can see that sample 1 is composed of monodisperse microspheres with a diameter of ca. 170 nm. However, dispersed nanocrystals are also observed around the microspheres. Figure 2d is the selected area electron diffraction (SAED) pattern of a single microsphere, revealing the single-crystal-like feature of the microsphere, indicating the oriented assembly of nanocrystals in each single microsphere. The SAED patterns of the individual aggregate constructed by the oriented organization of nanocrystals exhibiting single-crystal-like diffraction dots have been reported in the literature [11,33,34]. The energy dispersive spectroscopies (EDS) of microspheres (Figure 2e) and dispersed nanocrystals (Figure 2f) confirm that they both consist of Fe and O elements. The Cu peak is originated from the copper sample holder. The above information indicates that α-Fe_2_O_3_ microspheres are formed by the self-assembly of nanocrystals with diameters of several nanometers via an oriented aggregation mechanism. Since the microsphere is formed by oriented assembly of very small nanocrystals, the microsphere is characterized with a mesoporous structure, which is confirmed by nitrogen adsorption–desorption isotherm and the pore size distribution measurements, and this will be discussed below.

**Figure 3:**
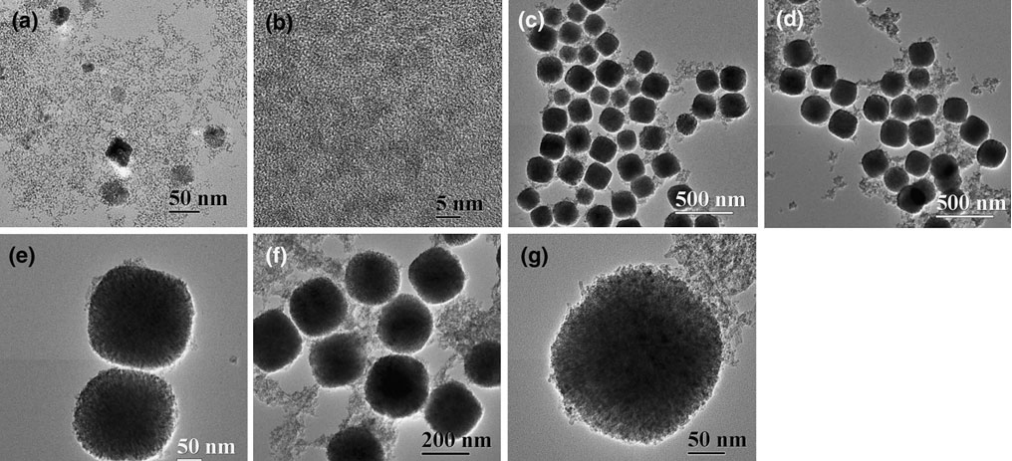
**TEM micrographs: a, b sample 2; c sample 3; d, e sample 4; f, g sample 5**.

We have found that sodium chloride (NaCl) plays an important role in the formation of monodisperse α-Fe_2_O_3_ mesoporous microspheres. Sample 2 was prepared in the absence of NaCl for comparison, and TEM micrographs of this sample are shown in Figure 3a, b. One can see that sample 2 prepared without NaCl consists mainly of very small dispersed nanocrystals with diameters of several nanometers and that particles formed by aggregation of nanocrystals are observed as a minor morphology, as shown in Figure 3a. The HRTEM image (Figure 3b) reveals that the average size of the nanocrystals is smaller than 5 nm. We propose that NaCl in the present synthesis acts as a promoter for the oriented assembly of α-Fe_2_O_3_ nanocrystals to form monodisperse mesoporous microspheres. It was reported that NaAc was used in the synthesis of Fe_3_O_4_ microspheres [32], and Ru, Pt and Rh particles [35-37]. In the present reaction system, NaCl may assist the complexation of PVP and iron ions, forming the monodisperse microspheres. We have also prepared sample 3 without using PVP, and the TEM micrograph is shown in Figure 3c. It can be seen that although α-Fe_2_O_3_ microspheres can be obtained without using PVP, the sizes of microspheres in sample 3 are not as uniform as those in sample 1 prepared in the presence of PVP. This result indicates that the addition of PVP is favorable for the formation of α-Fe_2_O_3_ monodisperse microspheres. Moreover, the concentration of PVP also influences the size of α-Fe_2_O_3_ monodispersed microspheres, which will be discussed below.

In order to control the size of mesoporous microspheres, comparative experiments were performed by changing the experimental parameters. Sample 4 was obtained with increased PVP concentration (0.222 g), while the other conditions were unchanged. One can see that sample 4 is also composed of α-Fe_2_O_3_ microspheres with an average diameter of ca. 205 nm, as shown in Figure 3d, e. Although sample 4 has a similar morphology to that of sample 1, the average diameter of microspheres in sample 4 is larger than that of sample 1, indicating that the concentration of PVP has an effect on the size of as-prepared α-Fe_2_O_3_ microspheres.

Figure 3f, g shows TEM micrographs of sample 5 prepared when the microwave-solvothermal time was increased from 30 to 60 min, and the average diameter of α-Fe_2_O_3_ mesoporous microspheres increases from ca. 170 to ca. 225 nm. This experimental result indicates that longer microwave-solvothermal time results in larger mesoporous microspheres. Thus, the size of α-Fe_2_O_3_ mesoporous microspheres can be controlled by adjusting microwave-solvothermal time.

**Figure 4:**
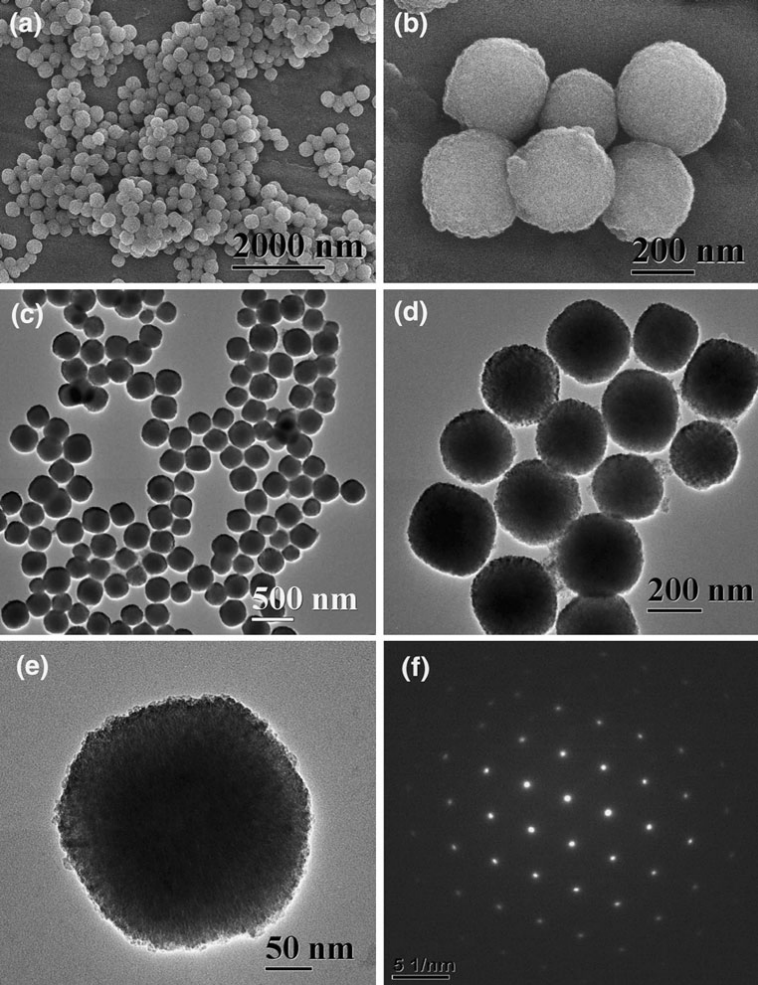
**Characterization of sample 6: a, b SEM micrographs; c–e TEM micrographs; f the SAED pattern of a single microsphere**.

Sample 6 was prepared at a higher microwave-solvothermal temperature of 140°C instead of 120°C, while the other conditions were kept unchanged. Figure 1b shows the XRD pattern of sample 6, from which one can see that the product is a single phase of α-Fe_2_O_3_ with a hexagonal structure (JCPDS No. 80–2377). The higher intensities of the XRD peaks of sample 6 compared with those of sample 1 (Figure 1a) indicate that the crystallinity of sample 6 is improved. Figure 4a–e shows the SEM and TEM micrographs of sample 6. One can see that almost exclusive α-Fe_2_O_3_ mesoporous microspheres assembled with nanocrystals are obtained and that dispersed nanocrystals are hardly observed compared with sample 1. However, the average diameter of α-Fe_2_O_3_ microspheres in sample 6 increases to ca. 260 nm, higher than that of sample 1 (170 nm), implying that higher microwave-hydrothermal temperature will produce α-Fe_2_O_3_ microspheres with larger size. Figure 4f shows the SAED pattern of a single microsphere, revealing the single-crystal-like feature of the mesoporous microsphere formed via an oriented aggregation of α-Fe_2_O_3_ nanocrystals. The above experimental results indicate that the size of α-Fe_2_O_3_ mesoporous microspheres can be controlled (in the range from ca. 170 to ca. 260 nm under the present experimental conditions used) by changing the experimental parameters such as the microwave-solvothermal time and concentration of PVP.

**Figure 5:**
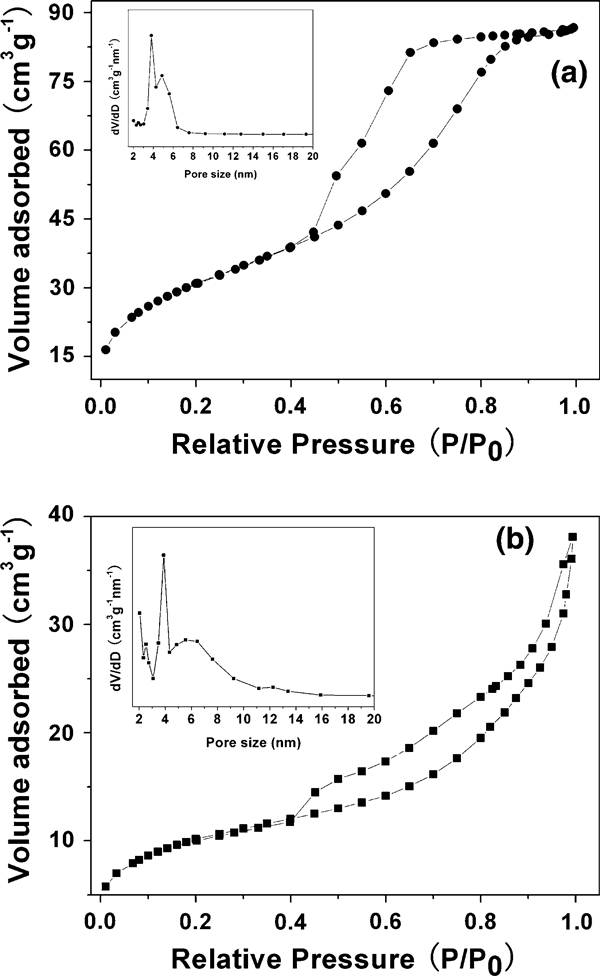
**Nitrogen adsorption–desorption isotherms and the pore size distributions of the as-prepared samples: a sample 1; b sample 6**.

We have measured the BET-specific surface areas and the pore size distributions of samples 1 and 6. Figure 5a, b shows the nitrogen adsorption–desorption isotherms and the pore size distributions of samples 1 and 6, which indicate that the BJH (Barrett–Joyner–Halenda) desorption average pore size and the BET-specific surface area are 4.3 nm and 114 m^2^/g for sample 1, and 7.9 nm and 37 m^2^/g for sample 6, respectively. Figure 5 indicates that there exist mesoporous structures in the α-Fe_2_O_3_ mesoporous microspheres. Sample 1 prepared at a lower temperature (120°C) has a much higher specific surface area and narrower pore size distribution than those of sample 6 prepared at a higher temperature (140°C). From the comparison of TEM micrographs of samples 1 and 6 (Figures 2, 4), one can see that the nanocrystals self-assembled in α-Fe_2_O_3_ mesoporous microspheres of sample 1 are smaller than those of sample 6. The oriented organization of smaller nanocrystals in α-Fe_2_O_3_ microspheres of sample 1 leads to smaller average pore size (4.3 nm); in contrast, the bigger nanocrystals in α-Fe_2_O_3_ microspheres of sample 6 result in larger average pore size (7.9 nm). On the other hand, the mesoporous microspheres constructed by the oriented organization of nanocrystals in sample 1 are much smaller (170 nm) than those of sample 6 (260 nm). These factors have effects on the BET-specific surface area, leading to the significant difference in BET-specific surface area between samples 1 and 6. These properties of α-Fe_2_O_3_ mesoporous microspheres will directly affect their photocatalytic activity, which will be discussed below.

**Figure 6:**
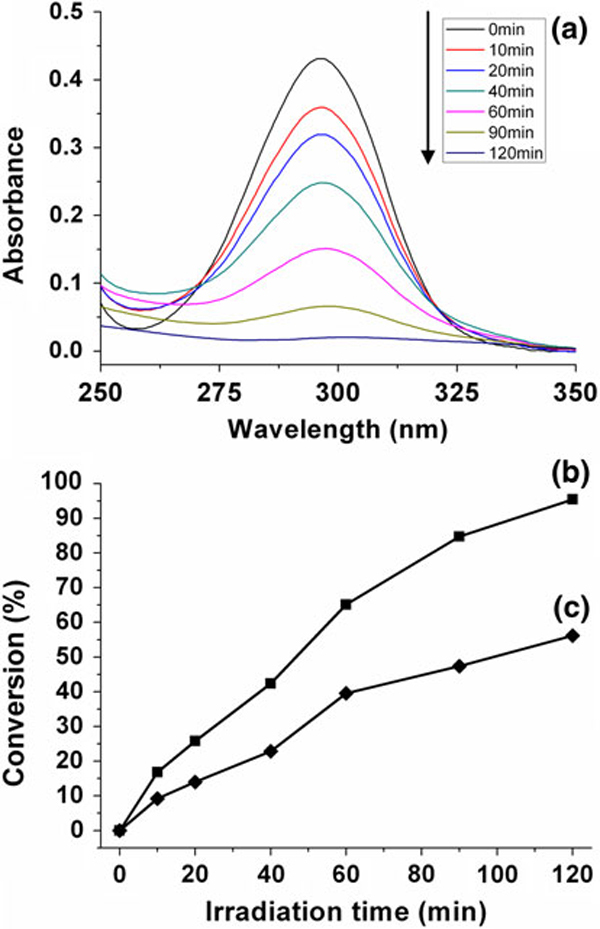
**a UV–vis absorption spectra of salicylic acid solution in the presence of sample 1 at different UV-irradiation times**. *b*, *c* The degradation percentage of salicylic acid with different as-prepared photocatalysts: *b* sample 1; *c* sample 6.

To evaluate the photocatalytic activity of α-Fe_2_O_3_ monodisperse mesoporous microspheres, the comparison experiments were performed. Figure 6a shows the UV–vis absorption spectra of salicylic acid solution in the presence of sample 1 at different UV-irradiation times, from which one can see that the concentration of salicylic acid decreases rapidly after UV irradiation. Figure 6b shows the degradation percentage of salicylic acid in the presence of sample 1, from which one can see that the degradation percentage of salicylic acid increases rapidly with increasing time and nearly complete in a time period of 120 min. The photocatalytic activity of sample 1 is much higher than that obtained in our previous work [11,12]. It can be found that the combination of the mesoporous structure with monodisperse microspheres is beneficial for the enhancement of the photocatalytic property of α-Fe_2_O_3_. We also investigated the photocatalytic activity of sample 6 as a reference. Sample 6 shows much weaker photocatalytic activity than sample 1, as illustrated in Figure 6c. It is obvious that α-Fe_2_O_3_ monodisperse mesoporous microspheres with higher specific surface area and narrower pore size distribution exhibit superior photocatalytic activity.

## Conclusions

We have developed a simple one-step NaCl-assisted microwave-solvothermal method for the preparation of α-Fe_2_O_3_ monodisperse mesoporous microspheres formed by oriented assembly of nanocrystals. In this approach, Fe(NO_3_)_3_ · 9H_2_O is used as the iron source, and PVP acts as a surfactant in the presence of NaCl in mixed solvents of H_2_O and ethanol. NaCl is found to play an important role in the formation of α-Fe_2_O_3_ monodisperse mesoporous microspheres. One of the advantages of this method is that the size of α-Fe_2_O_3_ mesoporous microspheres can be adjusted in the range from ca. 170 to ca. 260 nm by changing the experimental parameters. High photocatalytic activities in the degradation of salicylic acid are observed for α-Fe_2_O_3_ mesoporous microspheres. The combination of the mesoporous structure with monodisperse microspheres is beneficial for the enhancement of the photocatalytic property of α-Fe_2_O_3_ in the degradation of salicylic acid by giving α-Fe_2_O_3_ mesoporous microspheres higher specific surface area and narrower pore size distribution.
